# Efficacy and Safety of a Single Session of Micro‐Focused Ultrasound for Mid‐To‐Lower Facial Rejuvenation: A Retrospective Multimodal Assessment Study Using Clinical Evaluation, High‐Frequency Ultrasound, and VISIA Skin Analysis

**DOI:** 10.1111/jocd.71262

**Published:** 2026-11-13

**Authors:** Yuanyuan Qu, Fang Xiang, Jiawen Ma, Halina Hairola, Yuan Ding, Xiaojing Kang

**Affiliations:** ^1^ Department of Dermatology and Venereology People's Hospital of Xinjiang Uygur Autonomous Region; Xinjiang Clinical Research Center for Dermatologic Diseases; Xinjiang Key Laboratory of Dermatology Research Urumqi China

## Abstract

**Background:**

Micro‐Focused Ultrasound (MFU) is widely used for noninvasive facial rejuvenation. However, its effects on the middle and lower face have not been fully evaluated using multimodal assessment methods.

**Aims:**

This study aimed to evaluate the efficacy and safety of a single MFU treatment for tightening and lifting of the middle and lower face by combining clinical evaluation with imaging‐based assessments.

**Methods:**

Thirty female patients who underwent a single MFU treatment session were retrospectively included. Treatment was performed using both linear transducers (M3.0/M4.5) and large focal‐zone transducers (D3.0/D4.5). Clinical outcomes were evaluated using the Global Aesthetic Improvement Scale (GAIS), Facial Relaxation Scale (FRS), and Wrinkle Severity Rating Scale (WSRS) at baseline and at 1 and 3 months after treatment. Dermal thickness and echogenicity were assessed using high‐frequency ultrasound, and skin parameters were analyzed with the VISIA system. Pain scores and adverse events were also recorded.

**Results:**

GAIS and FRS scores improved significantly at both follow‐up visits compared with baseline (*p* < 0.001). Nasolabial fold severity was significantly reduced at 1 month (*p* < 0.0167), with no further significant change observed at 3 months. High‐frequency ultrasound showed significant increases in dermal thickness and echogenicity after treatment (*p* < 0.001). VISIA analysis demonstrated significant improvements in wrinkles, texture, pores, and brown spots (*p* < 0.0167), whereas spots, redness, and UV pigmentation showed no significant changes. Pain was mild in 70% of patients. All adverse events were mild and transient.

**Conclusions:**

A single MFU treatment can improve mid‐to‐lower facial laxity and overall facial appearance. Given the study limitations, this conclusion should be considered preliminary and exploratory in nature.

## 1 Introduction

In recent years, the demand for aesthetic procedures has increased markedly, with a growing preference for noninvasive approaches to facial rejuvenation in clinical practice [1]. With aging, laxity of the skin and soft tissues around the mandibular region gradually becomes more apparent, leading to a less defined jawline. The nasolabial folds deepen progressively, and marionette lines may begin to appear. These age‐related changes are mainly observed in the middle and lower face, an area that plays an important role in determining the perceived age of an individual.

Microfocused Ultrasound (MFU) can deliver energy precisely to deeper tissue layers, including the superficial musculoaponeurotic system (SMAS), deep dermis, and fibrous septa within the subcutaneous tissue. The thermal effect induces controlled microinjury in the target tissues, resulting in immediate contraction and tightening of collagen fibers, followed by gradual neocollagenesis over time. MFU is a specialized subdivision of high‐intensity focused ultrasound (HIFU) technology employed for precise facial rejuvenation. It utilizes micro‐focal zones and high‐frequency parameters to precisely target the SMAS and the dermis, distinguishing it significantly from the large‐focal zone, low‐frequency parameters characteristic of HIFU devices used for body contouring. MFU has been widely used for facial lifting because of its precision, short recovery time, and favorable safety profile [2, 3]. However, the mechanisms underlying its rejuvenation effects in the middle and lower face have not been fully clarified, and more objective evidence is needed to evaluate post‐treatment changes in skin appearance and tissue structure.

Therefore, we conducted a retrospective study to evaluate the efficacy and safety of a single MFU treatment for rejuvenation of the mid‐to‐lower face. A multidimensional assessment approach was adopted, including clinical grading scales, high‐frequency ultrasound imaging, and skin analysis using the VISIA system.

## 2 Materials and Methods

### 2.1 Patients

This retrospective study included 30 female patients who underwent facial rejuvenation treatment between March 2024 and March 2025. This retrospective clinical study was approved by the Institutional Review Board and the requirement for written informed consent was waived. All procedures were conducted in accordance with the Declaration of Helsinki. Patient data were anonymized before analysis.

The mean age of the patients was 35.43 ± 5.63 years. All patients had Fitzpatrick skin Types III‐IV and presented with signs of mid‐to‐lower facial aging, including skin laxity, increased fine lines, an indistinct jawline, and prominent nasolabial folds.

Patients were included if they met the following criteria: complete clinical data, including standardized photographs and evaluation records before and after treatment; absence of severe systemic diseases; and generally healthy facial skin condition.

Patients were excluded if they were pregnant or breastfeeding; had active facial skin diseases, infections, or inflammation, such as acne, eczema, or dermatitis; had received other facial rejuvenation procedures within the previous 6 months, including laser therapy, radiofrequency treatment, filler injection, or surgery; had coagulation disorders or were receiving anticoagulant therapy; were unable to tolerate ultrasound treatment; or had severe immune‐related or chronic diseases, such as uncontrolled hypertension or diabetes.

### 2.2 Methods

All patients underwent facial cleansing before treatment. Standardized photographs, VISIA skin analysis, and high‐frequency ultrasound examination were then performed. High‐frequency ultrasound imaging was conducted using a DUB Skin Scanner 75 system (TPM, Germany) with a center frequency of 22 MHz. Subsequently, each patient received a single MFU treatment session using the MFUS One system for rejuvenation of the mid‐to‐lower face.

Two categories of ultrasonic therapeutic transducers were employed in this study: linear‐focused cartridges and macro‐focused wide‐area cartridges. The linear‐focused cartridge series included M3.0 (focal depth: 3.0 mm) and M4.5 (focal depth: 4.5 mm). The M3.0 targets the deep dermis and superficial subcutaneous tissue, while the M4.5 targets the superficial muscular aponeurotic system (SMAS) fascia layer. The macro‐focused wide‐area cartridge series included D3.0 (focal depth: 3.0 mm) and D4.5 (focal depth: 4.5 mm). The pulse frequency was set to 8–10 Hz, and the device output power was set to 3.76–6.63 W. Output power is titrated for each patient based on three clinical indicators: facial subcutaneous fat thickness, the depth of the superficial musculoaponeurotic system (SMAS) fascia, and real‐time visual analog scale (VAS) pain feedback during treatment. For patients with thin subcutaneous tissues and a superficially located SMAS fascia, the lower power range (3.76–4.80 W) is selected; for those with a thick facial fat layer, a safe, medium‐to‐high power level within the device's compliant output range (4.81–6.63 W) is used.

Medical‐grade white marking pens were used to delineate mid‐to‐lower facial treatment zones, facial ligament attachment points, and key regions of skin laxity. All patients received standardized treatment following a fixed sequence: deep tissue layers were targeted before superficial layers, and linear‐focused cartridges were used prior to macro‐focused wide‐area cartridges. For linear‐focused cartridges, linear fixed‐point pulse delivery was performed within each pre‐delineated treatment grid: one focal pulse was emitted per site before moving to the adjacent site, with a 1‐mm interval between consecutive emission positions. For macro‐focused wide‐area cartridges, sliding overlapping application was performed in each predefined treatment zone, with progression to the next zone only after completion of the current zone. M4.5 and M3.0 linear focused applicators deliver 10 to 40 pulses per zone, with a cumulative total of 300 to 400 per patient for each linear focused applicator. The large focal zone wide‐area sliding applicator utilizes a sliding overlap technique within preset zones. Both the D4.5 and D3.0 applicators operate for 1 to 2 min per zone, with a total treatment duration of 14 to 16 min per applicator. For details on treatment zone division, linear applicator pulse counts, and large focal zone applicator operation duration, refer to Figure 1.

**FIGURE 1:**
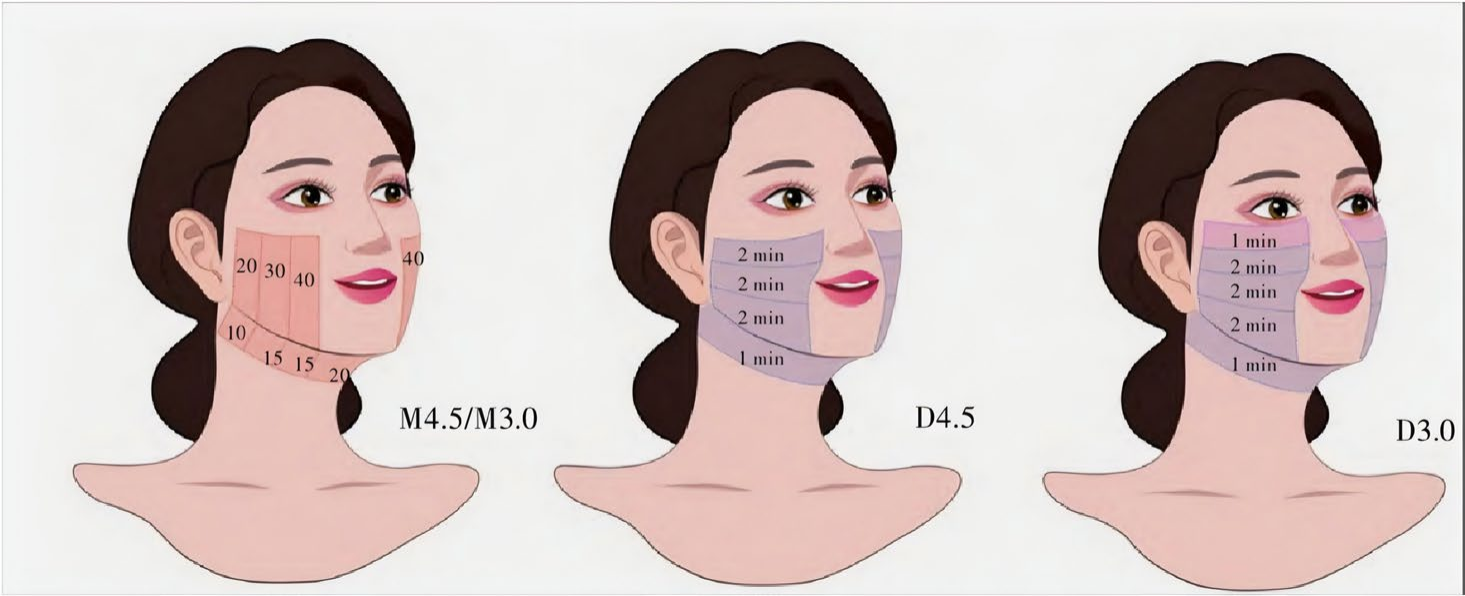
Treatment map. M4.5 and M3.0 indicate areas treated with the linear‐focused transducers, and the numbers on the diagram denote the number of treatment lines delivered to each area. D4.5 indicates areas treated with the 4.5‐mm large focal‐zone transducer, and D3.0 indicates areas treated with the 3.0‐mm large focal‐zone transducer. The numbers on the diagram indicate the treatment duration for each area.

After treatment, all patients were advised to enhance skin moisturization and maintain strict photoprotection. Follow‐up assessments were conducted at 1 and 3 months after treatment, including standardized photography and imaging examinations for comparative analysis (Figure 2). All treatment outcomes were assessed using a single‐blind method. Mask all basic patient information, test time identifiers, and original medical records before evaluation to avoid subjective evaluation bias. Two certified dermatologists who did not participate in case selection, clinical treatment, or original data collection independently completed imaging assessment and clinical efficacy evaluation, while incorporating patient self‐assessment results as supplementary evaluation evidence.

**FIGURE 2:**
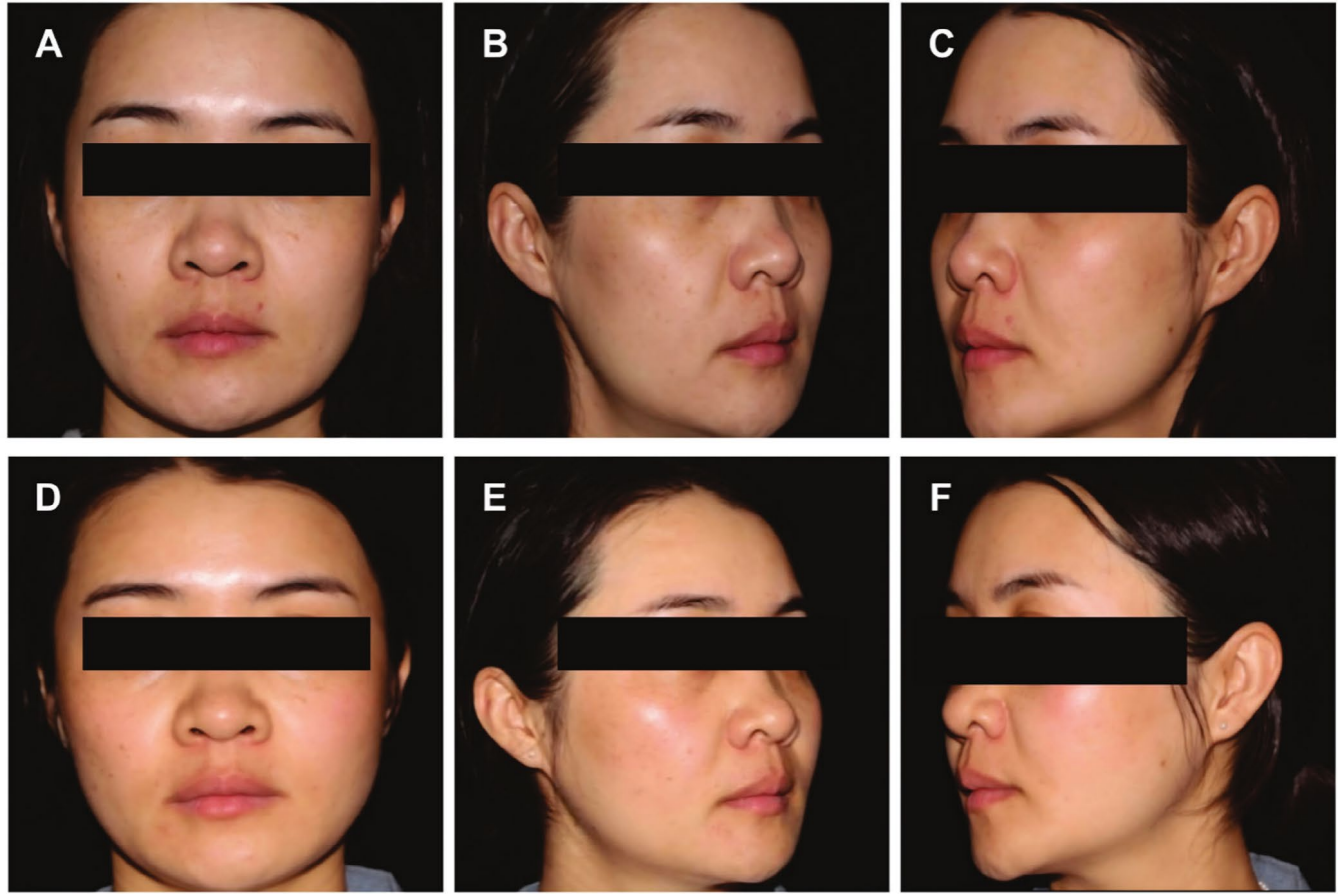
Representative clinical images of a patient before and 3 months after a single session of microfocused ultrasound (MFU) treatment for mid‐to‐lower facial rejuvenation. Panels (A–C) show the baseline images, and panels (D–F) show the images obtained 3 months after treatment. Panels (A) and (D) show frontal views, panels (B) and (E) show left lateral views, and panels (C) and (F) show right lateral views. Marked improvements in skin laxity and facial contour are observed at the 3‐month follow‐up compared with baseline.

### 2.3 Outcome Measures

#### 2.3.1 Overall Aesthetic Improvement

Treatment efficacy was assessed using the Global Aesthetic Improvement Scale (GAIS) [4] (Table 1). This was a retrospective longitudinal study where all cases were assigned a fixed baseline GAIS score of 2, indicating no aesthetic improvement. This approach established a standardized baseline to quantitatively assess facial improvements at 1 and 3 months postoperatively.

**TABLE 1:** Global Aesthetic Improvement Scale (GAIS).

1	Worse	Appearance is worse than before treatment.
2	No improvement	No visible change compared with before treatment.
3	Slight improvement	Appearance has improved slightly, but the change is not obvious.
4	Moderate improvement	Appearance has clearly improved, achieving the expected clinical effect.
5	Marked improvement	Appearance has improved markedly, exceeding the expected clinical effect.

#### 2.3.2 Facial Laxity Assessment

Mid‐to‐lower facial laxity was evaluated using the Facial Relaxation Scale (FRS) [5] (Table 2).

**TABLE 2:** Facial Relaxation Scale (FRS).

Score	Severity	Clinical description
0	None	The skin is firm. The jawline is clear, smooth, and well‐defined, with no loose tissue or sagging.
1–3	Mild laxity	Overall skin tightness begins to decline. Scores 1–2: The jawline changes from sharp to softer, but the contour remains clear. A slight decrease in skin elasticity may be observed during strong facial movement. Score 3: The jawline becomes more rounded. At rest, very mild submental skin laxity may begin to appear.
4–6	Moderate laxity	Laxity is clearly visible, with changes in facial contour. Score 4 (mild‐to‐moderate): The jawline becomes less defined. Early jowls may appear, with slight anterior displacement of soft tissue. Submental skin laxity becomes more noticeable. Score 5 (typical moderate): Jowls are clearly visible. The jawline becomes indistinct or partially lost. The nasolabial folds may become deeper and longer as the tissue descends. Score 6 (moderate‐to‐severe): Jowls become prominent and begin to sag. Soft tissue accumulates in the lower face, and the lower facial contour on frontal view begins to shift toward a triangular (or square) configuration. Neck laxity becomes apparent.
7–9	Severe laxity	Severe tissue ptosis with marked contour deformity. Score 7 (severe): The jowls show marked drooping, and the cervicofacial border becomes poorly defined. The lower face shows a triangular, mask‐like appearance. Clear neck folds are present. Score 8 (very severe): The jowls merge with the submental and neck tissues, creating a waterfall‐like sagging appearance. Marked skin laxity with deep folds is present. Score 9 (most severe): Extreme skin laxity and facial‐neck sagging are present. Normal facial landmarks are displaced, with widespread tissue ptosis.

#### 2.3.3 Nasolabial Fold Severity Assessment

The severity of nasolabial folds was graded according to the Wrinkle Severity Rating Scale (WSRS) [6] (Table 3).

**TABLE 3:** Wrinkle Severity Rating Scale (WSRS).

Score	Severity	Clinical description
1	Absent	No visible fold; continuous skin line.
2	Mild	Shallow but visible fold with slight indentation. The fold may or may not be visible at rest.
3	Moderate	Moderately deep fold, clearly visible at rest without facial expression, but disappears with gentle skin stretching.
4	Severe	Long and deep fold, clearly evident at rest. The fold improves with stretching but does not disappear completely.
5	Extreme	Extremely deep and long fold accompanied by facial sagging; very conspicuous at rest. Stretching provides minimal improvement. It may be associated with shadowing > 2 mm and a visible wrinkle line.

#### 2.3.4 High‐Frequency Ultrasound Assessment

High‐frequency ultrasound was used to measure dermal thickness and dermal echogenicity in the mid‐to‐lower face at baseline and at 1 and 3 months after treatment (Figure 3).

**FIGURE 3:**
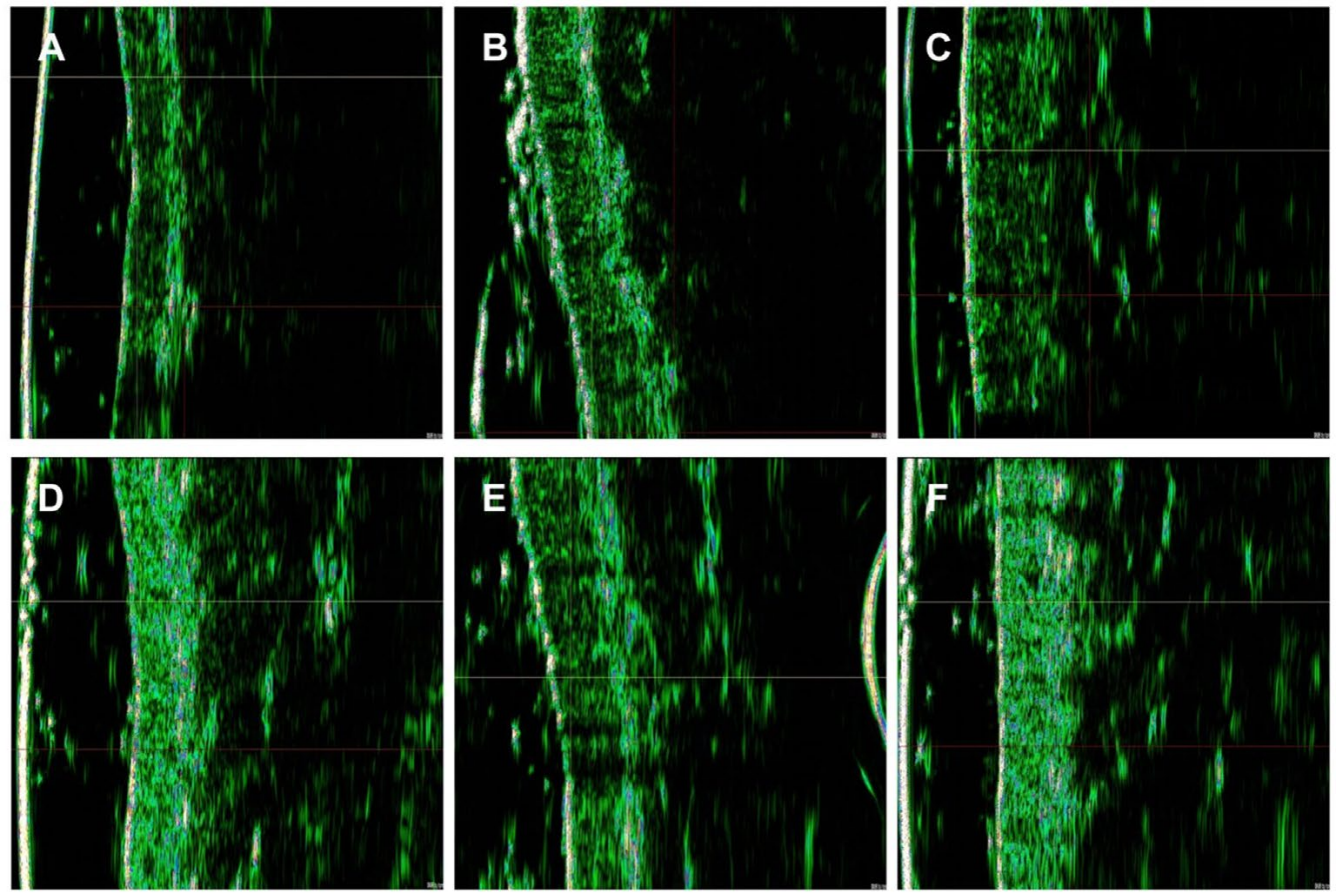
Representative high‐frequency ultrasound (HFUS) images of the mid‐to‐lower face showing changes in dermal thickness and dermal echogenicity before and 3 months after treatment in three patients. Panels (A–C) show the baseline HFUS images of three patients, and panels (D–F) show the corresponding images obtained 3 months after treatment. At the 3‐month follow‐up, all three patients showed increased dermal thickness and higher dermal echogenicity compared with baseline, indicating improvement in dermal structure after treatment.

#### 2.3.5 VISIA Skin Analysis

The VISIA system was used to assess skin parameters, including spots, pores, texture, wrinkles, and brown spots. Measurements were performed at baseline, 1 month, and 3 months after treatment (Figure 4).

**FIGURE 4:**
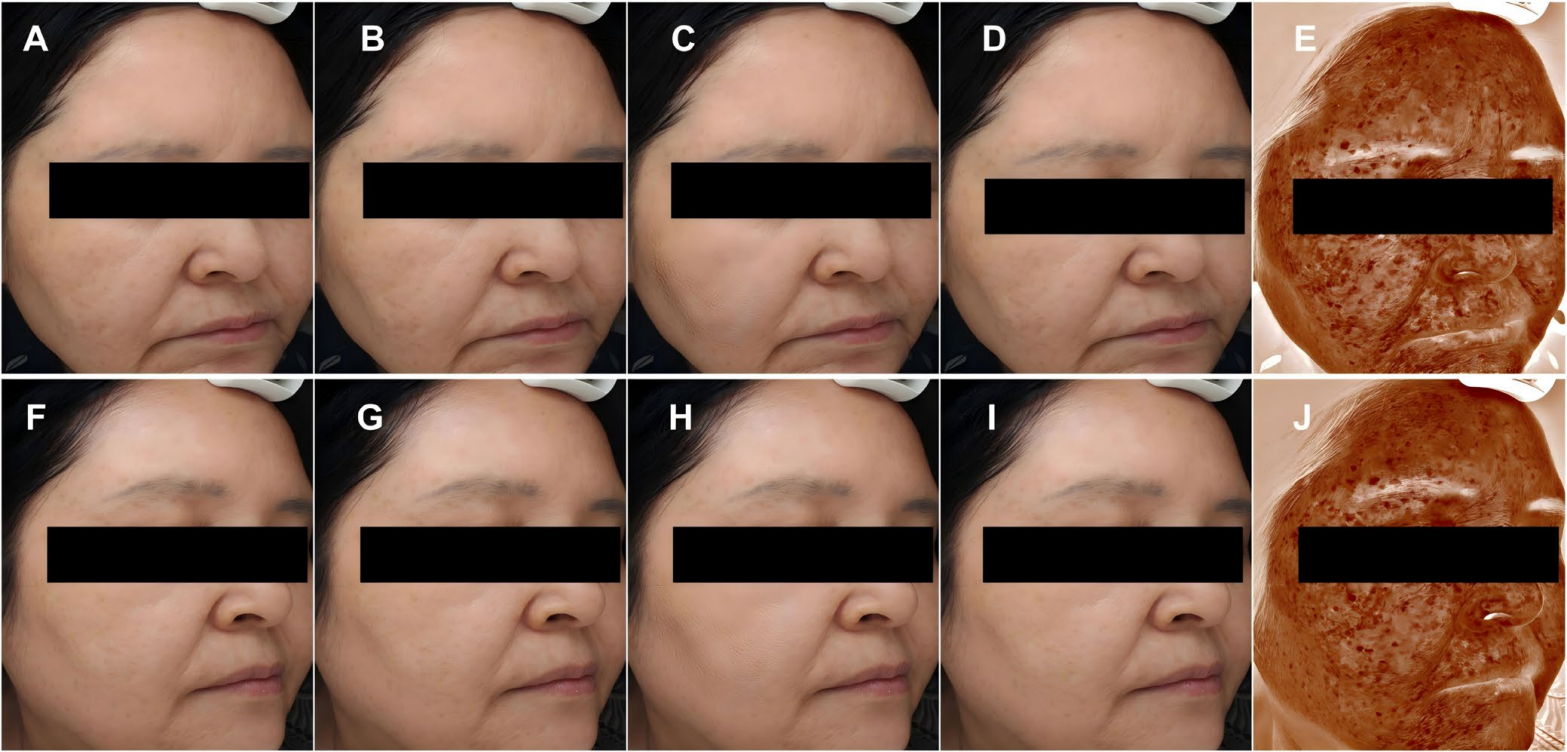
Standardized VISIA‐CR images of the mid‐to‐lower face from one patient before treatment and 3 months after treatment. Panels (A–E) show the baseline images, including pigmented spots, pores, skin texture, wrinkles, and brown spots, respectively. Panels (F–J) show the corresponding images obtained 3 months after treatment. The images demonstrate reductions in pigmentation, pores, and wrinkles, as well as smoother skin texture after treatment.

#### 2.3.6 Pain Assessment

Pain intensity during treatment was assessed using the Visual Analog Scale (VAS). Patients rated their pain on a scale from 0 to 10, with higher scores indicating greater pain intensity. Pain scores of 0–3 were classified as mild, 4–6 as moderate, and 7 or higher as severe.

#### 2.3.7 Adverse Events

Adverse events were monitored during treatment and throughout the follow‐up period. Events were recorded by both patients and physicians, including erythema, pain, swelling, and other treatment‐related reactions.

### 2.4 Statistical Analysis

Statistical analysis was performed using SPSS version 23.0. The Shapiro–Wilk test was applied to evaluate the distribution of continuous variables. Since all the continuous variables exhibited skewed distributions, data are expressed as median and interquartile range [M (P25, P75)]. Comparisons across multiple time points were performed using the Friedman test, followed by Wilcoxon signed‐rank tests with Bonferroni correction for pairwise comparisons. Categorical variables are expressed as numbers and percentages. A two‐sided *p* value < 0.05 was considered statistically significant.

## 3 Results

GAIS scores increased from a baseline median of 2 to 3 at 1 month and 3.5 at 3 months. FRS scores decreased from a baseline median of 4 to 3 at 1 month and remained at 3 at 3 months. WSRS scores decreased from a baseline median of 3 to 2 at both 1 and 3 months. The Friedman test showed significant differences across time points for all three scales (all *p* < 0.001). After Bonferroni correction, GAIS scores increased progressively over time, whereas FRS scores decreased progressively over time (all *p* < 0.0167). WSRS scores improved significantly at 1 month compared with baseline (*p* < 0.0167), but no significant difference was observed between 1 and 3 months (*p* > 0.0167) (Table 4).

**TABLE 4:** Comparison of GAIS, FRS, and WSRS scores before and after treatment (*n* = 30).

Variable	Statistic	Baseline	1 month	3 months	Friedman test	Wilcoxon test
GAIS	M (P25, P75)	2 (2, 2)	2.9 (3, 3)	3.5 (3, 4)	*χ* ^2^ = 48.86, *p* < 0.001	①vs.② *Z* = −4.874, *p* < 0.001^a^ ②vs.③ *Z* = −3.578, *p* < 0.001^a^ ①vs.③ *Z* = −4.736, *p* < 0.001^a^
GAIS	MR	1.10	2.18	2.72	*χ* ^2^ = 48.86, *p* < 0.001	
FRS	M (P25, P75)	4 (3, 4.5)	3 (3, 4)	3 (2, 3)	*χ* ^2^ = 45.871, *p* < 0.001	①vs.② *Z* = −4.796, *p* < 0.001^a^ ②vs.③ *Z* = −3.051, *p* = 0.002^a^ ①vs.③ *Z* = −4.919, *p* < 0.001^a^
FRS	MR	2.85	1.80	1.35	*χ* ^2^ = 45.871, *p* < 0.001	
WSRS	M (P25, P75)	3 (2, 3)	2 (2, 3)	2 (1.75, 2)	*χ* ^2^ = 29.429, *p* < 0.001	①vs.② *Z* = −3.606, *p* < 0.001^a^ ②vs.③ *Z* = −2.333, *p* = 0.02 ①vs.③ *Z* = −4.472, *p* < 0.001^a^
WSRS	MR	2.55	1.90	1.55	*χ* ^2^ = 29.429, *p* < 0.001	

*Note:* (1) For GAIS, higher scores indicate better improvement. (2) For laxity and wrinkle scores, lower scores indicate better improvement. (3) Time‐point comparisons were performed using the Friedman test. ① = baseline (before treatment); ② = 1 month post‐treatment; ③ = 3 months post‐treatment.

^a^ Indicates a statistically significant difference in the Bonferroni‐corrected Wilcoxon test (*p* < 0.0167).

Dermal thickness increased from a baseline median of 1844.0 μm to 1846.7 μm at 1 month and 1853.85 μm at 3 months. Dermal echogenicity increased from a baseline median of 13.75 to 15.65 at 1 month and 18.65 at 3 months. The Friedman test showed significant differences across time points for both variables (both *p* < 0.001), and pairwise comparisons demonstrated progressive increases over time (Table 5).

**TABLE 5:** Comparison of facial dermal thickness and dermal echogenic density before and after treatment (*n* = 30).

Variable	Statistic	Baseline	1 month	3 months	Friedman test	Wilcoxon test
Dermal thickness (μm)	M (P25, P75)	1844.0 (1648.50, 2018.50)	1846.70 (1651.38, 2018.75)	1853.85 (1661.98, 2030.78)	*χ* ^2^ = 58.067 *p* < 0.001	①vs.② *Z* = −4.763, *p* < 0.001^a^ ②vs.③ *Z* = −4.783, *p* < 0.001^a^ ①vs.③ *Z* = −4.782, *P* < 0.001^a^
Dermal thickness (μm)	MR	1.03	1.97	3.00	χ^2^ = 58.067 *p* < 0.001	
Dermal density (%)	M (P25, P75)	13.75 (11.23, 16.28)	15.65 (12.38, 18.25)	18.65 (15.03, 21.85)	*χ* ^2^ = 53.664 *p* < 0.001	①vs.② *Z* = −4.710, *p* < 0.001^a^ ②vs.③ *Z* = −4.764, *p* < 0.001^a^ ①vs.③ *Z* = −4.763, *p* < 0.001^a^
Dermal density (%)	MR	1.05	2.02	2.93	*χ* ^2^ = 53.664 *p* < 0.001	

*Note:* (1) Time‐point comparisons were performed using the Friedman test. ① = baseline (before treatment); ② = 1 month post‐treatment; ③ = 3 months post‐treatment.

^a^ Indicates a statistically significant difference in the Bonferroni‐corrected Wilcoxon test (*p* < 0.0167).

VISIA analysis showed significant improvements in wrinkles, texture, pores, and brown spots over time (all *p* < 0.0167). In contrast, changes in spots, red areas, and ultraviolet spots were not statistically significant (Table 6).

**TABLE 6:** Comparison of VISIA scores before and after treatment (*n* = 30).

Variable	Statistic	Baseline	1 month	3 months	Friedman test	Wilcoxon test
Spots	M (P25, P75)	34.10 (31.40, 37.40)	33.08 (30.21, 36.79)	32.86 (27.64, 38.93)	*χ* ^2^ = 14.067, *p* < 0.001	①vs.② *Z* = −2.355, *p* = 0.019 ②vs.③ *Z* = −2.026, *p* = 0.043 ①vs.③ *Z* = −2.149, *p* = 0.032
Spots	MR	2.50	1.97	1.53	*χ* ^2^ = 14.067, *p* < 0.001	
Wrinkles	M (P25, P75)	10.71 (7.54, 22.03)	9.05 (5.70, 18.30)	6.21 (3.63, 11.75)	*χ* ^2^ = 57.513, *p* < 0.001	①vs.② *Z* = −4.782, *p* < 0.001^a^ ②vs.③ *Z* = −4.703, *p* < 0.001^a^ ①vs.③ *Z* = −4.782, *p* < 0.001^a^
Wrinkles	MR	3.00	1.98	1.02	*χ* ^2^ = 57.513, *p* < 0.001	
Texture	M (P25, P75)	3.21 (2.20, 4.56)	2.54 (1.98, 3.61)	2.22 (1.41, 3.15)	*χ* ^2^ = 38.400, *p* < 0.001	①vs.② *Z* = −4.535, *p* < 0.001^a^ ②vs.③ *Z* = −3.980, *p* < 0.001^a^ ①vs.③ *Z* = −4.206, *p* < 0.001^a^
Texture	MR	2.80	2.00	1.20	*χ* ^2^ = 38.400, *p* < 0.001	
Pores	M (P25, P75)	10.75 (7.00, 15.08)	10.73 (6.32, 13.83)	9.40 (6.23, 14.85)	χ^2^ = 17.067, *p* < 0.001	①vs.② *Z* = −2.458, *p* = 0.014^a^ ②vs.③ *Z* = −2.417, *p* = 0.016^a^ ①vs.③ *Z* = −2.437, *p* = 0.015^a^
Pores	MR	2.53	2.00	1.47	*χ* ^2^ = 17.067, *p* < 0.001	
Red areas	M (P25, P75)	26.83 (24.56, 29.52)	26.37 (24.21, 28.68)	25.85 (22.94, 29.67)	*χ* ^2^ = 6.667, *p* < 0.05	①vs.② *Z* = −2.252, *p* = 0.024 ②vs.③ *Z* = −1.573, *p* = 0.116 ①vs.③ *Z* = −1.903, *p* = 0.057
Red areas	MR	2.33	2.00	1.67	*χ* ^2^ = 6.667, *p* < 0.05	
Brown spots	M (P25, P75)	39.46 (32.50, 44.86)	37.41 (31.98, 44.04)	35.23 (31.18, 43.29)	*χ* ^2^ = 20.467, *p* < 0.001	①vs.② *Z* = −3.240, *p* < 0.001^a^ ②vs.③ *Z* = −2.067, *p* = 0.039 ①vs.③ *Z* = −2.808, *p* = 0.005^a^
Brown spots	MR	2.60	1.97	1.43	*χ* ^2^ = 20.467, *p* < 0.001	
UV spots	M (P25, P75)	16.80 (14.80, 26.75)	17.32 (14.21, 25.62)	18.21 (13.55, 22.86)	*χ* ^2^ = 1.400, *p* > 0.05	/
UV spots	MR	2.17	1.97	1.87	*χ* ^2^ = 1.400, *p* > 0.05	

*Note:* (1) Time‐point comparisons were performed using the Friedman test. ① = baseline (before treatment); ② = 1 month post‐treatment; ③ = 3 months post‐treatment.

^a^ Indicates a statistically significant difference in the Bonferroni‐corrected Wilcoxon test (*p* < 0.0167).

Pain scores indicated mild pain in 21 patients (70%) and moderate pain in nine patients (30%). No patient reported severe pain, and no topical anesthetic was applied before treatment.

Adverse events were mild and self‐limited. Transient erythema, edema, stinging, and numbness were observed, whereas no allergic reactions, bruising, facial expression abnormalities, facial volume loss, or muscle atrophy were recorded (Table 7).

**TABLE 7:** Adverse events after treatment.

Adverse event	Cases, *n* (%)	Duration
Facial erythema	6 (20.0)	1 h
Mild facial swelling	4 (13.3)	2 day
Facial stinging	13 (43.4)	5–7 day
Cheek numbness	8 (26.7)	10–14 day

## 4 Discussion

Facial rejuvenation treatments are increasingly developing toward noninvasive, precise approaches with shorter recovery times [7, 8]. This study discusses the application of MFU for tightening and lifting of the mid‐to‐lower face and evaluates its effects on skin structure and appearance from a clinical perspective.

In this study, GAIS scores increased significantly at 1 and 3 months after treatment, while FRS scores decreased significantly. These findings indicate that MFU treatment can effectively improve mid‐to‐lower facial laxity and enhance the overall youthful appearance of the face. The clinical improvement may be associated with the mechanism of MFU and the treatment strategy of the device used in this study. The device used in this study was the Peninsula MFUS One system, which adopts a treatment mode combining a linear‐focused transducer and a large focal‐zone transducer. The linear‐focused transducer precisely delivers ultrasound energy to the SMAS, forming small thermal coagulation points. This directly induces collagen denaturation and contraction in the SMAS layer, known as the immediate contraction effect of the fascia layer, which provides the mechanical basis for early tightening and lifting. As a complementary approach, the large focal‐zone transducer distributes energy more evenly within the deep dermis and fibrous septa of the subcutaneous tissue, providing broad and low‐dose thermal stimulation over a larger area. Therefore, this combined treatment mode enables layered and precise thermal stimulation from the deep fascia to the dermal layer, while minimizing thermal injury to non‐target tissues and improving the therapeutic effect [9]. This treatment also activates an early inflammatory and repair response in the tissue. The controlled thermal injury generated during treatment triggers an early inflammatory response, thereby activating fibroblasts. The results of this study support this mechanism, as dermal thickness and dermal echogenicity increased significantly in a time‐dependent manner after treatment. The increase in dermal echogenicity may reflect increased collagen fiber content and a denser arrangement of dermal fibers [10]. Therefore, MFU may initiate collagen regeneration and remodeling within 3 months after treatment. The subsequent synthesis and reorganization of new collagen and elastin may further consolidate and enhance the early contraction effect.

In the present study, WSRS scores for nasolabial folds improved significantly at 1 month after treatment, with no further significant decrease from 1 to 3 months. Therefore, for fine lines mainly associated with skin laxity and soft‐tissue sagging, the tightening effect induced by MFU appears to be effective in the early stage. Subsequently, more sustained and substantial improvement may result from gradual dermal collagen remodeling. To further improve deeper static wrinkles, longer follow‐up or combination treatment with fillers may be considered.

Based on the current relevant literature, MFU, as a common noninvasive approach for skin rejuvenation, is often used in combination with neuromodulators, fillers, and other injectable treatments in clinical practice. Its long‐term safety has been confirmed by large‐sample studies [11]. More importantly, there is clear evidence that the treatment effect of MFU is influenced by patients' baseline conditions. Excessive skin laxity and BMI > 30 may be regarded as relative contraindications. The greater the degree of skin laxity and BMI, the lower the treatment efficacy [12]. Therefore, the treatment plan should be formulated according to patient suitability.

VISIA skin analysis can objectively reflect the condition of the epidermis and superficial skin layers. Therefore, the improvements in wrinkles, texture, pores, and brown spots after treatment suggest that MFU may have beneficial effects on skin texture, smoothness, and partial pigment uniformity in addition to deep lifting. The improvements in skin texture and pores may be related to epidermal renewal through the post‐treatment repair mechanism and enhanced dermal collagen support. The reduction in brown spot scores may be associated with pigment redistribution after mild treatment‐induced inflammation and changes in the overall light‐reflecting characteristics of the skin. These mechanisms have been supported by previous literature. Pan et al. further reported [13] the effects of MFU in improving ultraviolet‐induced pigmentation and pigment spots, and indicated that MFU could also improve skin firmness and brightness. These findings suggest that MFU/HIFU‐type ultrasound technology may have the potential for ultrasound‐based skin rejuvenation. In addition, the therapeutic effect of MFU may be enhanced when combined with topical preparations. A clinical trial on the synergistic effect of focused ultrasound and topical preparations showed that the vibration and thermal effects generated by focused ultrasound could promote transdermal absorption of topical preparations and enhance their local efficacy [14]. In that study, MFU combined with topical glutathione and hyaluronic acid was significantly superior to topical preparations alone in improving fine wrinkles, pigmentation, and skin water content (all *p* < 0.05). This provides important evidence for MFU‐based combination treatment. It is worth noting that spots, red areas, and UV spots did not change significantly in this study, which is consistent with the technical characteristics of MFU, as its main target is collagen rather than melanin or blood vessels. Therefore, for patients whose main concerns are pigment spots or telangiectasia, combined treatment with targeted modalities such as pulsed light or laser may be considered. Wang et al. [15] further reported that MFU combined with delicate pulsed light (DPL) was safe and effective for facial rejuvenation and was superior to single treatment in improving skin tone, providing an example for clinical combination treatment.

The safety and tolerability findings showed that no topical anesthesia was used during treatment. Nevertheless, 70% of patients reported only mild pain, and 30% reported moderate pain, indicating excellent overall tolerability. It is noteworthy that patients with relatively abundant facial subcutaneous fat appeared to experience less pain during treatment. Two possible hypotheses may explain this finding: first, subcutaneous adipose tissue may have a buffering effect on the delivered energy; second, the density of nerve endings may differ among the selected treatment regions. Strict control of the focal depth of energy is fundamental to treatment safety, as it helps avoid injury to the deep facial nerve and its branches. This has been supported by an ultrasound measurement study published in 2022 [16], which confirmed that soft‐tissue thickness differs among various lower facial regions, including the preauricular area, lower cheek, and mandibular region, and is influenced by factors such as age, sex, and facial contour. In older patients, male patients, and individuals with thinner facial contours, the superficial musculoaponeurotic system may be located more superficially. Therefore, when formulating an individualized treatment plan, it is necessary to consider the anatomical and individual characteristics of facial tissues. For areas with thinner subcutaneous fat and a shallower superficial musculoaponeurotic system, energy parameters should be appropriately reduced, the focal depth should be adjusted, and adequate pain management should be provided to optimize the treatment experience. Post‐treatment adverse reactions were mild and transient, mainly including temporary erythema, edema, tingling, and numbness, which resolved spontaneously within a few days. No severe or permanent adverse reactions were observed. These findings are highly consistent with the favorable safety profile of MFU reported in the current literature [9, 17].

This study has several limitations. The sample size was small, and all patients were female. The 3‐month follow‐up period was insufficient to evaluate long‐term efficacy and adverse reactions. In addition, this was a single‐center retrospective study without a control group. Therefore, large‐sample, prospective, randomized controlled studies are needed to further verify the conclusions and optimize treatment parameters. More importantly, the present study only observed changes in the dermis, and the complete mechanism underlying the lifting effect of MFU has not been fully clarified.

Given the aforementioned limitations, this study suggests a potential association between a single session of MFU treatment and improvements in mid‐to‐lower facial skin laxity and overall appearance; however, these findings should be interpreted as preliminary and exploratory.

## Author Contributions

Y.Q. conceived and designed the study, as well as wrote the manuscript; F.X. and H.H. performed data collection and follow‐up; J.M. completed the image processing. Y.D. and X.K. reviewed and approved the final version of the manuscript. All authors contributed to manuscript revision, read, and approved the final submitted version.

## Funding

The authors have nothing to report.

## Ethics Statement

This study was conducted in strict accordance with the ethical principles of the Declaration of Helsinki. It was approved by the Ethics Committee of the People's Hospital of Xinjiang Uygur Autonomous Region (approval no: KY2026030301). All patients were fully informed of the study purpose, treatment procedures, and potential risks, and provided written informed consent. This is a retrospective observational study. All data were anonymized to protect patient privacy strictly and were used only for the analysis of this study.

## Conflicts of Interest

The authors declare no conflicts of interest.

## Data Availability

The data that support the findings of this study are available from the corresponding author upon reasonable request.
